# Understanding the risk perception of visceral leishmaniasis exposure and the acceptability of sandfly protection measures among migrant workers in the lowlands of Northwest Ethiopia: a health belief model perspective

**DOI:** 10.1186/s12889-022-13406-3

**Published:** 2022-05-16

**Authors:** Resom Berhe, Mark Spigt, Francine Schneider, Lucy Paintain, Cherinet Adera, Adane Nigusie, Zemichael Gizaw, Yihenew Alemu Tesfaye, Dia-Eldin A. Elnaiem, Mekuriaw Alemayehu

**Affiliations:** 1 Department of Health Education and Behavioral Sciences, University of Gondar, College of Medicine and Health Science, Institute of Public Health, Gondar, Ethiopia; 2 Department of Family Medicine, CAPHRI School for Public Health and Primary Care, Maastricht University, Maastricht, The Netherlands; 3 Department of Health Promotion, School for Public Health and Primary Care (CAPHRI), Maastricht University, Maastricht, The Netherlands; 4 Department of Disease Control, Faculty of Infectious and Tropical Diseases, London School of Hygiene & Tropical Medicine, London, UK; 5 KalaCORE Country Office, Addis Ababa, Ethiopia; 6 Department of Environmental Health and Safety, Institute of Public Health, College of Medicine and Health Sciences, University of Gondar, Gondar, Ethiopia; 7 Department of Social Anthropology, Faculty of Social Sciences, Bahir Dar University, Bahir Dar, Ethiopia; 8 Department of Natural Sciences, University of Maryland Eastern Shore, Princess Anne, MD USA

**Keywords:** Health belief model, Perception, Leishmaniasis, Qualitative research

## Abstract

**Background:**

Visceral leishmaniasis (VL) is the leading cause of health concerns among Ethiopian migrant workers. Understanding risk perception and health-protective behavior are significant challenges in the prevention and eradication of the disease. As a result, studies are required to assess these important epidemiological factors, which will provide guidance on how to assist migrant workers in taking preventive measures against VL.

**Method:**

We conducted qualitative research among migrant workers on seasonal agricultural farms in Northwest Ethiopia between June and November 2019 to assess their perception of the risk of contracting VL and their willingness to use protective measures against the disease. Seventeen focus group discussions and 16 key informant interviews were conducted to study migrant workers’ risk perception in relation to sandfly bite exposure and use of sandfly control measures. For analysis, all interviews were recorded, transcribed, and translated. ATLASti was used to perform qualitative content analysis on the data.

**Result:**

Migrant workers are fearful of VL because of previous exposure and the disease’s prevalence in the area. They believe, however, that VL is a minor illness that is easily treated. While Insecticide Treated Nets (ITNs) are widely accepted as a protective measure, there are still reservations about using them due to the seasonality of the transmission, difficulties in hanging them on farm areas, and a preference for alternative traditional practices. Regardless of perceived self-efficacy, the central cues were the message delivered by the health workers and an increase in sandfly bite irritation. Based on the findings, three levels of intervention modalities are suggested: 1) increasing pre-arrival awareness through outdoor media (posters, stickers, billboards), 2) encouraging proper use of protective measures upon arrival at farm camps, and 3) informing departing workers on disease recognition and best practices for health-seeking continuous use of protective measures at home.

**Conclusion:**

This finding suggests that VL prevention interventions should focus on individuals’ perceptions in order to promote consistent use of protective measures. The findings are highly useful in planning effective interventions against VL.

**Supplementary Information:**

The online version contains supplementary material available at 10.1186/s12889-022-13406-3.

## Background

Visceral leishmaniasis (VL), also known as kala-azar, is a vector-borne disease caused by the protozoan parasite Leishmania donovani (Order Kinetoplastida: Family Trypanosomatidae) and spread by phlebotomine sand flies (Order Diptera: Family: Psychodidae). When individuals get VL, the most common symptoms are fever and, in some cases, enlargement of the spleen and liver [1, 2]. This disease is the world’s second-leading parasitic killer (after malaria) [1, 3]. It is endemic in 62 countries, with 200 million people at risk [4]. Despite being a neglected tropical parasitic disease, it is estimated that 500,000 cases of VL occur each year [4, 5], with a prevalence of 2.5 million [6]. If left untreated, the mortality rate from VL is nearly 95% [5, 7].

Currently, East Africa carries the highest burden of VL with 57% of the global cases [8]. The disease is endemic in Ethiopia, Kenya, Somalia, Sudan, and Uganda, where severe epidemics have killed a large number of people. VL is common in Ethiopia’s lowlands of the south and southwest, as well as the Metema-Abuderafie agricultural fields of the northeast [9–11]. The vector of the disease, Phlebotomus orientalis, thrives in *Acacia seyal*-*Balanites aegyptiaca* vegetation that grows on Black Cotton soil in the northwest and most parts of VL endemic sites in Ethiopia [11].

VL is frequently found in remote or difficult-to-access areas where health services are scarce or non-existent. As a result, those most likely to be infected are primarily disadvantaged populations with little understanding of disease transmission [7, 12, 13]. Therefore, the geographic distribution of VL in Africa is associated with low socioeconomic status, poor socio-cultural practices, and lack of access to health services [12, 14–16]. Furthermore, massive rural-urban migration and agro-industrial projects that bring non-immune urban dwellers into endemic rural areas have an impact on VL epidemiology [17].

Migrant workers are at high risk of contracting VL, owing to the deplorable and harsh conditions under which they work on farms [9, 18–20]. Between June and November each year, up to 500,000 migrant workers, primarily from the surrounding Amhara and Tigray highland areas, visit the Metema-Abuderafie lowlands for weeding and reaping of sesame, sorghum, and cotton [9, 21]. It is also believed that when migrant workers return to the highlands, they spread the infection [22]. A good example is the endemic VL foci in the Libo Kemkem and Fogara districts, where a severe epidemic of the disease resulted in 2450 primary cases between 2003 and 2005 [9].

Despite the fact that migrant workers in the lowlands of Northwest Ethiopia account for more than 60% of the disease load, no control measures are in place to reduce pathogen transmission to these vulnerable populations [9, 23]. Furthermore, a significant impediment to effective VL control is a lack of information on migrant workers’ knowledge, attitude, and risk perception, which affects their exposure to sandfly bites and the acceptability of vector control tools.

There is little known about the level of VL knowledge, health perception, and socioeconomic and behavioral factors that influence migrant workers’ exposure to sandfly bites, as well as the acceptability and use of vector control tools [9]. Several studies on the knowledge, attitudes, and practice of VL have been conducted in Kenya [12], the Republic of South Sudan [14], India [15], and northwest Ethiopia [16]. The researchers reported a number of elements related to perception, attitude, knowledge, and behavior toward the disease in these studies. The authors also found a link between the KAP of VL and a variety of factors such as education level, socioeconomic status, age, gender, housing, and resting behavior. Several human practices, such as deforestation and moving during the evening hours when the sand fly is active, were found to play important roles in the transmission of VL [24]. These studies, however, were limited to resident populations and did not address seasonal and migrant workers’ exposure to sandfly bites or their acceptance of control tools. Furthermore, most studies failed to use a theoretical framework to guide research on VL perception [25]. The current study aims to use a theoretical framework to assess Ethiopian seasonal and migrant workers’ perceptions of sand fly bite exposure and their acceptance of sandfly control measures. This research also provides a slew of intervention strategies that could supplement ongoing disease-control efforts. The findings of this qualitative study will be especially useful for policymakers and program implementers interested in developing appropriate VL intervention programs in Northwest Ethiopia and other East African regions. The following research questions were addressed in the study: Do seasonal and/or migrant workers practice VL prevention (e.g., sand fly control)? Do they believe they are vulnerable to VL (due to a lack of sand fly control measures)? Do workers believe that contracting VL has negative consequences (posing a serious health risk)? Furthermore, do the workers believe that the benefits of engaging in protective behavior (reducing VL risk) outweigh the costs (money spent on vector control tools)? How do they respond to information indicating that they are at risk, and how do they perceive their ability to use available sand fly control measures?

### Application of health belief model constructs to VL behavioral epidemiology

The Health Belief Model (HBM) was developed in the 1950s by social psychologists in the United States Public Health Service to explain individuals’ pervasive and ubiquitous failure to participate in projects to prevent and recognize disease [26, 27]. Individuals are expected to avoid disease and practice healthy behavior if they accept that doing so will protect them from contracting the disease. As determinants of health behavior, the HBM distinguishes six types of risk perception: perceived susceptibility, perceived severity, perceived benefits, perceived barriers, cues to action, and self-efficacy.

For the better part of a century, the HBM has been used to predict health-related behaviors and improve interventions to change behaviors. Previous literature review studies have shown that the HBM is useful in anticipating and clarifying cancer screening and HPV immunization [28, 29]. Because of its natural conceptualizations, its modifiable beliefs have become popular for use in interventions. HBM components are also frequently used in tailored interventions to change people’s beliefs [24]. For example, if people do not see the benefits of an activity or action, the intervention should help them see the benefits in the first place. Several studies have found that interventions tailored to specific barriers predict adherence to recommended health behaviors [30, 31]. According to research, perceived susceptibility to illness is an important predictor of preventive health behaviors [32]. Perceived barriers to healthy behaviors, in particular, are the most powerful predictor of whether people are willing to engage in healthy behaviors [33]. In fact, in recent years, self-efficacy has been identified as one of the most important factors in an individual’s ability to successfully use protective tools [34].

The HBM is being used for the first time to assess the perception of VL risk and the acceptability of sandfly control measures against the disease. Conducting qualitative research guided by theoretical frameworks can provide valuable in-depth insights into migrant workers’ perceptions of leishmaniasis prevention and control, thereby improving our understanding of existing quantitative data on migrant views, perceptions, and experiences [35, 36].

## Methods

To report the findings, we used the COREQ (Consolidated Criteria for Reporting Qualitative Research) method. In addition, a checklist is provided as additional information (see Supplementary file 1).

### Research team

Our multidisciplinary research team included four behavioral epidemiologists, three behavioral scientists, and three environmental health researchers. The fieldwork was directed by the project coordinator, an entomologist with advanced training in qualitative research methods. The research team was also assisted by public health experts from the University of Gondar and respondents’ known farmland owners.

### Study design and setting

A qualitative phenomenological study was conducted to investigate VL risk perceptions and acceptability of sand fly control measures among seasonal and migrant workers in agricultural farms in NW Ethiopia.

The research was carried out in seasonal farms in Abdurafi (West Armachiho area) and Metema, which are located in the North Gondar administrative zone of Amhara regional state, about 250 and 165 km north of Gondar town, respectively. Lemma et al. (2014) [18] and Mekuriaw et al. (2019) [9] describe the ecology of the study area. The year is divided into two seasons: the rainy season (June–October) and the dry season (November–May). The hottest month is May, and the wettest month is August. Balanites aegiptiaca trees can be found at about 25 m intervals in any direction in the Abdurafi and Metema lowlands’ typical agricultural fields. The open spaces between these trees are commonly used for sesame cultivation. Labor migrants worked to remove weeds from sesame seedlings after the land was plowed and seeded in mid to late June, mostly after settling in the agricultural fields. The sesame field is weeded again during the flowering stage, around August. During harvest season in September and October, the same labor migrants harvest and separate the seed from the plant before returning to their home in the highlands. Between June and November each year, up to 500,000 migrant workers, primarily from the surrounding Amhara and Tigray highland areas, visit the Metema-Abuderafie lowlands for weeding and reaping of sesame, sorghum, and cotton [9, 21].

According to the 2007 population and housing census report, West Armachio Woreda has a total population of 35,486 people, with 19,517 men and 15,969 women. According to the same census report, the total population of Metema district is 110,231 people, with 58,734 men and 51,497 women. Both areas have basic health care services. The Abdurafi inpatient kala-azar treatment center (run by the international non-governmental organization Medicines Sans Frontières) in Abdurafi provides medical care to patients suffering from Leishmaniasis, HIV-VL coinfection, and snakebite. Metema Hospital’s kala-azar treatment center offers outpatient and inpatient medical services for Leishmaniasis patients, as well as HIV-VL coinfection and a variety of other hospital-level services.

### Study sample and participants

To recruit study participants for Focus Group Discussions (FGDs) and Key Informant Interviews (KIIs), a purposeful sampling technique was used. The sample size was not determined prior to the start of the study. As a result, we continued FGDs and KIIs until we reached saturation, at which point no new information was provided [36–38]. There were 16 KIIs and 17 FGDs in total. Participants in the focus group were male migrant workers who came to the Metema-Abuderafie lowlands from the surrounding Amhara and Tigray highlands to weed and harvest sesame, sorghum, and cotton from June to November. Due to harsh conditions and cultural issues, the number of women involved in farming is negligible.

The 17 FGDs were attended by a total of 187 migrant workers. Sixteen KII participants included government officials, farm owners, farm managers, migrant worker leaders (Koberary), and health professionals. Some of the migrant workers stayed in the lowlands for one to 2 years during the previous or subsequent dry season, without establishing residency. These were distinct from seasonal workers, who are highland residents who come to the lowlands for one agricultural season only (June to October) and do not stay during the dry season (November to May).

### Inclusion criteria

Eligible seasonal and migrant workers who met the following criteria: (a) they were identified as migrant workers/seasonal workers by their farm landowners and Koberay (according to the criteria defined above); (b) they were 18 years or older; (c) they were not agricultural laborers who are residents of the study area (i.e., a person who has lived in the lowlands area for more than 3 years and has a home/address in one of the villages; (d) they were confirmed by the health professional and Koberary as mentally capable of taking part in this study, and (e) they were Amharic speaker.

### Recruitment

Through a farmland labor administrative system, potential eligible migrant workers were identified by their farmland owners. This system recorded the names and contact information of migrant workers who participated in weeding and harvesting during the summer and dry seasons. The study was announced to eligible workers by their “Koberary” (leader of the migrant worker). A study information sheet and a study consent form were included in the consent package. Migrant workers who agreed to take part in the study returned the consent form to the research team via Koberary. On the consent form, migrant workers were required to indicate their contact information and preferred contact times.

### Data collection instruments

Expert researchers and well-trained research assistants involved in other KalaCORE consortium epidemiological studies gathered the qualitative data. Prior to the surveys, the research team attended a three-day workshop led by professional behavioral epidemiologists on how to conduct FGDs and KIIs. During the KII and FGD, we used a semi-structured topic guide that included important information about the ecology and control of the VL vector [39]. Topics included VL knowledge, perception, behavior, and the use of preventive measures. The guides were written in English and then translated into Amharic (the local language of the participants). Discussions among the research team helped to shape the perception questions. Questions probed migrant workers’ perceptions of susceptibility [40], seriousness, benefits [32], and barriers, as well as action cues [41]. We incorporate the concept of self–efficacy [42] to improve its predictive capacity.

The semi-structured guide was used to conduct one-on-one interviews with key informants and focus groups with migrant workers. The KII interviews and FGD discussions were held in quiet locations with adequate privacy. These locations included tea houses, open farmland shelters, farmer’s and migrant workers’ homes, and farmland owners’ offices. One researcher carried out KIIs. During the focus group discussions, researchers worked in pairs, with one serving as moderator and the other as note taker. All KIIs and FGDs were held in Amharic. The interviewers met after each round of KIIs and FGDs to discuss and take notes on the main findings and potential difficulties. This step provided an opportunity to remind study staff about the objectives of the interviews and specific topics for the next round of interviews. It also aided in the development of new topics and the adaptation of related questions.

All KIIs and FGDs were digitally recorded with the participants’ permission. The participants were informed that all information would be coded to protect privacy and confidentiality. Interviews and focus group discussions were conducted until all categories were well defined and saturated. Before conducting the study, the research team reviewed all study materials and pilot-tested them.

### Data analysis

The interviewers and other accredited Ethiopian interpreters transcribed and translated each recorded interview and FGD. In addition, the text passage was read by members of the research team to acclimate themselves to the data and set up the task of codes and classification. A qualitative content analysis approach was subsequently used to identify themes and investigate critical factors influencing migrant workers’ risk perception regarding exposure to sandfly bites and the use of vector control tools (ITNs).

To minimize bias and ensure that all significant codes were captured, the coding cycle began with a conventional, inductive qualitative content analysis. The transcripts were initially read line by line. Their content was then examined, contrasted, and classified by using a summarization name (a “code”) that depicted what was interpreted as important in the passage. The codes were then grouped around the HBM domains to create more abstract classifications [34]. In this sense, a classification is a group of codes that share a commonality [43]. If a code could not be linked to any of the domains, a new classification was assigned to those domains to ensure that all information is captured and that they fit in the current model. This aided us in thoughtfully validating and broadening the underlying theoretical framework [44]. We created strings of meaning across classifications in light of the emerging classifications. Following that, we analyzed latent and shown content, and each interview was chosen as the unit of analysis [43].

### Ethical considerations

This study was ethically approved by the institutional review board (IRB) of the University of Gondar and the Health Education Department. The objectives and details of the study were explained to eligible study participants. Those who agreed to the study were accepted after signing a written informed consent. Participants were informed of their right to withdraw from the study at any time and to have their data excluded from analysis. During the study, any suspected cases of VL were referred to a nearby health center for proper diagnosis and treatment. To protect data privacy, information was identified using codes rather than participants’ names. Hard copies of questionnaires were kept safely in the Principal Investigator’s (PI) office. Any electronic files were kept on a computer that was password-protected.

## Results

### Characteristics of the study population

Between June and October 2019, key informants and migrant workers were interviewed. Seventeen focus group discussions (FGDs) with 8–12 migrant workers were held, with a total of 187 participants from 11 farms (large and small). The FGD participants’ mean (±SD) age was 23 (±4.66) years. Their education ranged from no formal education to secondary school. All of the participants in the focus group discussions were male migrant workers. A total of 16 individual interviews were also conducted. The mean (±SD) age of the interviewees was 31 (±6.75) years. More than half of the participants had tertiary or secondary education (see Table 1). Due to the harsh conditions and cultural issues, the number of women who participate in farming activities is negligible.

**Table 1:** Socio-demographic characteristics of participants of Focus group discussion and interviews in Metema -Abuderafie, 2019

FGD participants			Interview participants		
Characteristics	N	%	Characteristics	N	%
Total	187	100		16	100
**Gender**			**Gender**		
Male	187	100	Male	15	93.7
Female			Female	1	6.3
**Age (mean (SD)**			**Age (mean (SD)**		
23 (±4.66)			31(±6.75)		
**Type of participants**			**Type of participants**		
Students	55	29.4	Health office heads	2	12.5
Farmers	114	60.9	Health extension worker	1	6.3
Forest workers	7	3.7	Malaria and vector born officer	1	6.3
Milisha	8	4.4	Public health officers	1	6.3
Others	3	1.6	Farm (owners, managers, and leaders)	11	68.6
**Ethnicity**			**Ethnicity**		
Amhara	171	91.4	Amhara	10	62.5
Tigray	13	7.0	Tigray	6	37.5
Oromo	3	1.6	Oromo		
**Religion**			**Religion**		
Orthodox Christian	178	95.2	Orthodox Christian	15	93.7
Muslim	9	4.8	Muslim	1	6.3
**Leishmaniasis endemicity at the permanent resident**			**Leishmaniasis endemicity at the permanent resident**		
Low land endemic areas	20	10.7	Low land endemic areas	11	68.7
High land endemic areas	167	89.3	High land endemic areas	5	31.3
**Educational status**			**Educational status**		
Non formal education	82	43.9	Non formal education	1	6.3
Formal education	105	56.1	Formal education	15	93.7

### The health belief model (HBM) constructs

**Fig. 1:**
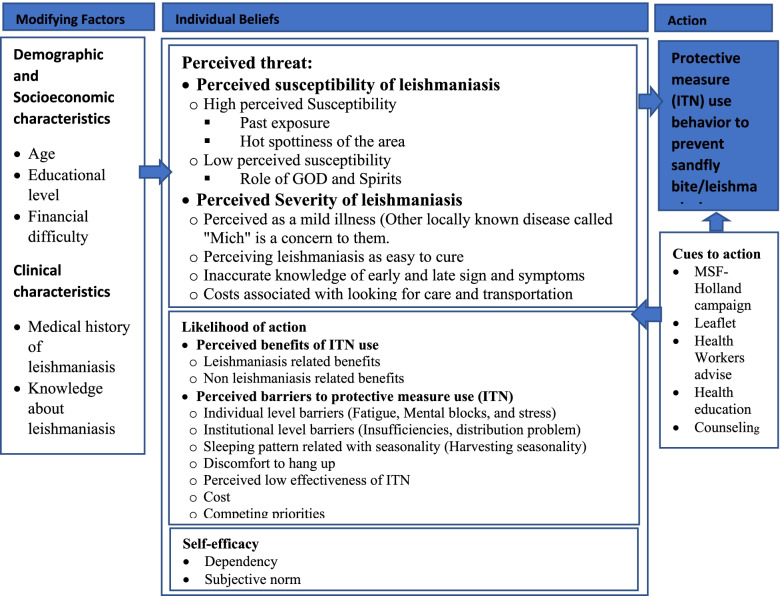
Results of Focus group discussion and key informant interviews. *Note.* ITN=Insecticide treated net

The results for the chosen HBM constructs, perceived susceptibility and seriousness, perceived barriers and benefits, self-efficacy, and cues to action, are shown below. Participants’ verbatim statements are provided in detail for each construct. In general, study participants regarded VL as a low threat to their well-being. Although beliefs that VL can be prevented by using an ITN increase the likelihood of action, actual utilization of ITNs was disabled by a number of factors, including seasonality of transmission, cost, individual and institutional barriers, inconvenience, perceived ineffectiveness, lack of awareness, ITN insufficiency, competing priorities, and the belief that the advantage can be achieved through the use of traditional methods. The migrant workers rarely expressed a sense of self-efficacy and stated that despite health awareness messages delivered by Medicins Sans Frontières (MSF-Holland) health extension workers, the main cue for them to use an ITN was increased sandfly bite irritation. Fig. 1 depicts each component of the HBM.

### Perceived susceptibility and seriousness of VL

The participants were almost unanimous in their vulnerability to VL, with little variation in their responses. Participants who had previously had VL or were aware of it in other people were more likely to believe that they were vulnerable to VL than other workers who had no prior experience with the disease. Furthermore, many migrant workers stated that VL is a concern for them due to what they have heard about the disease’s prevalence in the area. The migrant workers reported that the sandfly lives in their workplace and has bitten them. They did not, however, mention any negative health effects.

*“ We’ve heard that previous workers suffered from sandflies bite...We are prone to sandfly bites, and as a result, it will be irritating...We are vulnerable to sandfly bites because we work in an area where sandflies thrive, and we prepare food and sleep anywhere in the farmland.”(FGD, Migrant workers, from large and small farmland areas).*

On the other hand, many participants perceived lower risk of VL disease and were unconcerned about sandfly bites. This was much more evident in the migrant workers’ FGDs during the weeding and harvesting season. These workers attributed the disease to supernatural forces rather than sand fly bites: *“God, not the ITN, prevents leishmaniasis, and it is caused by a spirit.” (FGDs of migrant workers from small and large farmlands).*

Despite the fact that the migrant workers believed they were susceptible to sandfly bites or VL, the severity of the disease was deemed minor. One migrant worker put it succinctly: *“Almost everyone realizes they are vulnerable; however, no one takes it seriously.”* Many participants saw VL as a minor disease that, if treated promptly and appropriately, can be cured. Instead, an illness locally known as *“Mich”,* inexactly deciphered as “sunstroke”, was viewed as a more severe issue than VL. Because of the hot weather and the risk of contracting the disease *“Mich”*, almost all migrant workers spend the night in an open field inside the farm during harvesting season.

*“ Nowadays... when some of the workers experience some kind of sign and symptom, they suspect malaria and we send them to the health center for treatment, but when they arrive at MSF, they are told they have Kala-azar....we don’t know about Kala-azar”. (KII, HEW*).

Another effect of VL perceived by migrant workers was the costs associated with seeking care. When a migrant worker became ill, the economic consequences included spending money on transportation, diagnosis, and treatment, as well as missed workdays.

### Perceived benefit and barrier to protective measure usage (ITN)

Our study participants acknowledged the benefit of using an ITN as a protective measure against VL. During the sesame weeding season, sleeping in *“Gebaza”* (a straw and grass sub-shelter where migrant workers sleep) and staying warm under the ITN was advantageous. According to some of our participants, the number of sandflies is higher during the sesame weeding season, which influences both the perceived intensity of VL and sandfly bite irritation. Sandfly bite irritation influenced the perceived added benefit of the ITN as sandfly bite protection.

**Fig. 2:**
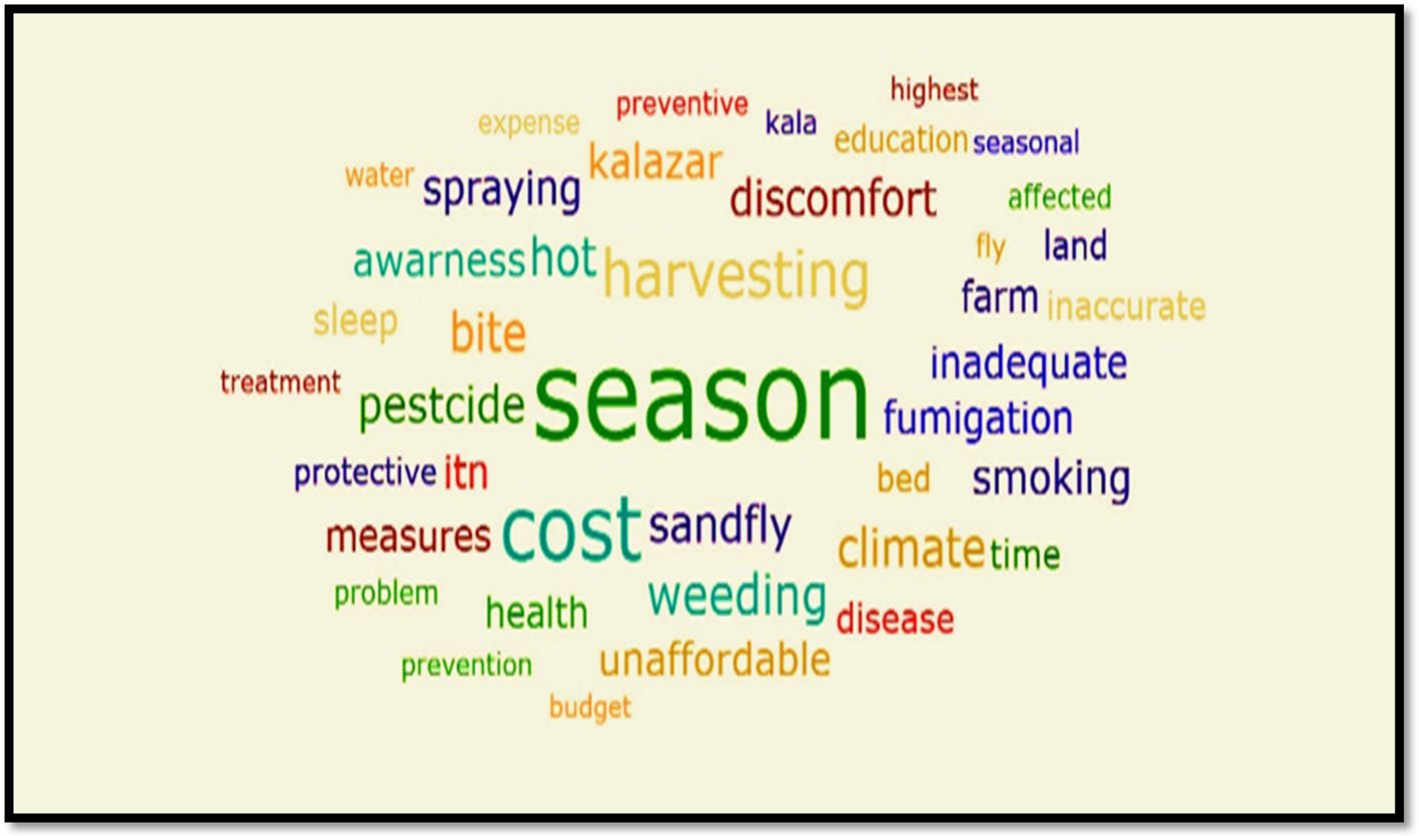
Visual representation (word cloud) of a perceived barrier for utilization of protective measures

Our participants mentioned a variety of barriers to ITN use against sandfly bites. The most significant barriers to ITN use in farmland areas are listed in Table 2 and illustrated visually in Fig. 2. The most commonly reported barrier to ITN use was that workers found them difficult to hang on their sleeping grounds in farmland areas. Some participants stated that they did not use ITN because it was inconvenient due to their communal and outdoor sleeping arrangements on the farms. Because of the daytime heat and humidity during the harvesting season, almost all migrant laborers work in open fields inside the farm during the cooler nights. ITN use is clearly prohibited during these late-night activities. Even when workers are tired and want to sleep at night, their motivation to hang and use ITN is reduced. Migrant workers discussed “absence of rest” or “feeling exhausted” as crucial reasons for not hanging and sleeping under an ITN.

*“.... we are aware of the advantages of using a bed net.” Therefore, we brought a few bed nets to the farm, and we always kept them inside our bags. The ITN is difficult to install, especially during harvesting season, when we work through the night inside the farms”. (FGD, migrant worker, from large farmland).*

**Table 2:** Top reasons for non-consistent protective measures (ITNs) from free listing and ranking activity in Metema -Abuderafie, 2019

Participants	Abuderafie district		Metema district	
	Large Farmland areas	Small farmland areas	Large Farmland areas	Small farmland areas
**Migrant workers**	Seasonality	Seasonality	Seasonality	Fumigation, spray
	Mental barriers	Discomfort to hang	Inconvenience	Lack of awareness
	Discomfort to hang	Cost	Distribution problem	Cost
	Inadequacy of net	Mental block	Ineffectiveness	Fatigue
	Fatigue	lack of awareness	Cost	Spraying
	Awareness problem	Stress	Insufficiency	Distribution problem
**Farmland owners and managers**	Inadequacy of net	Seasonality	Seasonality	Heat
	Lack of awareness	fumigation sprays.	Ineffectiveness	Laziness
	Ineffectiveness	Laziness	Lack of awareness	Difficulty in hanging
**Health workers**	Lack of awareness		Lack of awareness	
	Seasonality		discomfort to hang it	
	Discomfort to hang it		fumigation	
	fumigation		Stress (mental barriers)	
	Fatigue or laziness		Cost	
**Farm leader (Koberary)**	Seasonality	Seasonality	Insufficiency	ineffectiveness
	Fatigue or laziness	Fumigation,sprays	Cost	Laziness
	Discomfort to hang	Discomfort to hang	Seasonality	Lack of awareness

The seasonality of activities and behavior was another major factor that reduced continued ITN use. These working and resting behaviors, according to the participants, varied greatly between harvesting and weeding seasons. Sleeping under a net, for example, was perceived as extremely difficult due to the heat during the harvesting season, a barrier that appeared to be critical for the participant’s ITN use. The ITN, on the other hand, was perceived to protect against sandfly bites during weeding seasons. According to the HBM, this is a reasonable illustration of how perceived benefits and barriers are weighed against one another [45].

*“...Even if we wanted to use the bed net, it is too hot and difficult to hang on the farm.*

*area, and Gebaza......” (FGD, migrant workers from a large farmlands.)*

The previously mentioned belief in other causes of VL also contributed to aversion to using ITNs. Aside from sandfly, VL was thought to be caused by sexual activity, open defecation, filthy drinking water, bad food, and ravenousness. Eliminating water ditches, eating healthy foods, and focusing on cleanliness were all ways to prevent these causes. Concurrently, some migrant workers announced that they were using fumigation (burning wood or tires) to control sandflies in and around their sleeping areas. As a result, non-proven protective measures may be used incorrectly [46]. As a result of these beliefs, the workers did not see the need for the prevention behaviors such as sleeping under an ITN:

*“We cannot prevent this by using bed nets..... We can avoid it by smoking, using traditional herbal remedies, and not sleeping on the ground...all of these can help protect us from the bite “.. (FGD, migrant workers, large and small farmland areas).*

*“VL is caused by sexual activity...poor hygiene, sanitation, and eating bad food.” We prevent leishmaniasis by eating healthy foods and drinking clean water.” (FGD, migrant workers, large and small farmland areas).*

Although all farmland owners/managers stated that they received free ITNs from the government to distribute to migrant workers, it was felt that these ITNs were insufficient. Furthermore, many migrant workers stated that their limited access to ITNs was due to specific corruption in how ITNs are distributed. At the district health office level, these include bribery, mismanagement, and partiality. A few respondents blamed health authorities for collecting ITNs only to sell them later to landowners or market vendors who buy and sell them at exorbitant prices. Furthermore, the cost of ITN was mentioned as a barrier to ITN ownership, as 150 Ethiopian Birr (5 dollars) for an ITN was viewed as unaffordable when migrant workers think to buy one for themselves from the market:

*“The government freely distributes ITN... However, the owners give us some of them...There are also rumors of distribution issues...the bed net is not distributed evenly...it is given to whomever your favorite person is... They also sold it to businessmen...We are eager to have sandfly bite protective tools [ITN], but the cost of the material must be reasonable.” (FGD, M.W., from large farmland).*

Despite the fact that VL disease is common and the consequences are severe, none of the key informants reported that prevention and control are given top priority. According to a key informant, approximately 200,000 ETB (Ethiopian Birr) ($6061) is budgeted for insecticide spraying to prevent and control malaria.

*“We don’t have a budget or a plan to prevent and control leishmaniasis..... Other diseases, such as onchocerciasis, are funded by non-governmental organizations. We have set aside up to 200,000 ETB for malaria control, primarily for spraying “.. (KII, a member of the District Health Office, and HEW, Farm owners).*

### Self-efficacy and cues to action

Despite high perceived self-efficacy, there was a strong reliance on the government to provide the ITN or other protective measures. When migrant workers needed to discuss the risk of VL and the use of protection with their partners or supervisors, efficacy was also an issue. Many migrant workers, for example, stated that if a farm manager/owner insisted, they would use ITN. Furthermore, external influence from friends, farm leaders, and managers was found to be more valuable in developing self-efficacy or persuading people to use protective measures (ITN).

The workers’ increased irritation from sandfly bites was the primary cue for them to use protective measures (ITN). MSF-HOLLAND staff members, village health extension workers, farm owners, managers, and leaders were also encouraged to use ITN or other protective measures.

### Modifying factors

There was a significant disparity in educational backgrounds, with a few participants claiming to be illiterate. From various perspectives, the educational level appeared to alter the decision-making process. All migrant workers thought that being educated made it easier to use protective measures. Sandfly bites are linked to VL, according to a few educated key informants. Most participants stated that financial constraints in paying for their education, fertilizer, and food costs prevented them from considering purchasing ITN or other protective measures against sandfly bites.

### Intervention modality

**Fig. 3:**
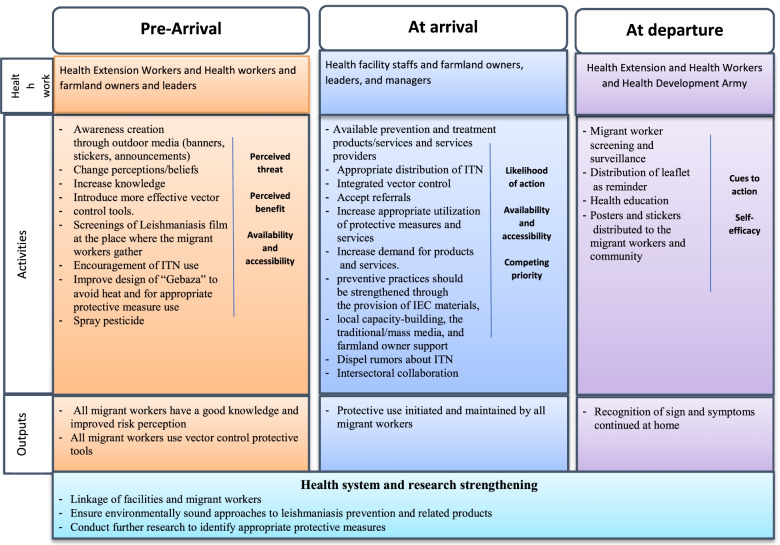
Three-level intervention modalities for prevention and control of Leishmaniasis among migrant workers

We propose that the intervention against VL transmission among migrant workers be carried out at three stages in relation to the timing of migrant workers’ movements between their home areas and the farmlands: pre-arrival, arrival, and departure (Fig. 3).

1. Pre-arrival - the goal at this level is to raise awareness through outdoor media (posters, stickers, billboards) and screening in order to correct misconceptions about the true causes of VL and its transmission. Although a few migrant workers were aware that sandflies cause VL, this did not prevent them from believing in other potential causes. As a result, comprehensive scientific education campaigns should be implemented to educate migrant workers about the transmission and why sandflies only transmitted VL, and “GOD” will do so through these insects. This will necessitate learning how to increase the use of protective measures as well as ensure the early identification of sick migrant workers through clinical screening upon their arrival at farms so that they can be effectively and promptly referred to a treatment center.

2. At-arrival– The goal of intervention at the arrival level is to encourage the use of protective measures by farmworkers throughout their stay. The most frequently mentioned barrier to ITN use was that they were uncomfortable and difficult to hang on sleeping grounds in farmland areas. As a result, we must find local solutions to install the nets. Furthermore, ITNs that are self-supporting, available in a variety of sizes, and do not take up a lot of space may be more convenient for workers to use. Furthermore, as the demand for products and services grows, it is recommended that the cost of ITN be reduced in order for it to be widely used. This could be accomplished through mass purchasing, local tailoring, community-based dissemination mechanisms, and public appropriation. Wearing long sleeves at night and using repellents such as mosquito loops, moisturizers, and sprays should also be part of integrated VL control strategies. This will necessitate learning how to ensure that all migrant workers receive ITN or other protective measures as soon as possible and that those migrant workers can continue to use ITN or other protective measures when they travel to farmland areas or *Gebaza*.

3. At-departure – The goal at the departure level is to inform travelers about the recognition of VL signs and symptoms, as well as the continued use of protective measures once they arrive in their home countries. Because the majority of social cues are learned from peers, health care providers should use the peers of migrant workers to disseminate prevention information. This will require learning how to recognize the symptoms of VL, the need for prompt treatment-seeking, and the importance of continued use of ITN.

## Discussion

Visceral leishmaniasis is a major contributor to the country’s disease burden among migrant and seasonal workers in NW Ethiopia. Despite advances in understanding the epidemiology of VL in Ethiopia, control of transmission of VL among this vulnerable population remains a challenge. This is due in part to a scarcity of information on the disease’s behavioral epidemiology. In this study, we used a health belief model as a framework to assess migrant and seasonal workers’ perceptions of the risk of VL and the acceptability of sand fly control measures in agriculture farms in NW Ethiopia. Overall, our study found that workers had a negative perception of VL. As a result, this perception was linked to inconsistency in the use of sand fly control measures. A number of barriers were clearly contributing to the poor perception of VL risk, including an underestimation of the disease’s severity as “mild” and an overestimation of their susceptibility to the infection due to their beliefs that “The VL is from God,” with no specific cause. We discuss different constructs of the HBM as they apply to VL perception and acceptability of sand fly tools among workers in separate subheadings below. However, due to the workers’ lack of VL knowledge, as previously described (9), the researchers were unable to delve into additional details about the perceived benefit, cues to action, self-efficacy, and moderating factors in the study group.

### Perceived susceptibility and severity

The workers reported being vulnerable to VL because of their risky night sleeping habits and food preparation in the shade of Acacia and Balanitis trees. In this way, the workers’ perceptions are somewhat accurate in light of the known epidemiology of VL in Ethiopia [39] and the behavior of the sandfly vector, which is associated with Acacia and Balanites trees in Metema-Abuderafie lowland farm areas [47]. Surprisingly, workers with prior VL experience had improved VL risk perception and a proclivity to use sand fly protection measures. This observation is consistent with previous research demonstrating the importance of experiential components in individual decision-making [36, 37]. However, the decision to use preventive measures by these and other workers was influenced by a number of factors, including a strong belief that “Almighty God” is responsible for their health. Indeed, a portion of the migrant workers appeared to rely on God or spirit to recover from illness. Some migrant workers believed that, aside from God, there was nothing they could rely on to prevent VL. This finding suggests a lower perceived vulnerability, which may lead to a lower likelihood of taking precautions.

Our extensive conversations with migrant workers taught us that VL is regarded as a minor illness and is not a major health issue on the farms. This low perception was caused in part by the long incubation period of VL [48], which means that those who contract the infection on the farms will not experience symptoms of the disease until they return to their homeland several months later. As a result, farm workers will no longer recognize VL as a common disease. Instead, because of the distorting skin results, another disease caused by sunstroke, locally known as “*Mich*”, was viewed as a more serious issue than VL. In contrast to some studies [30, 49–52], our findings supported the belief that VL was not recognized as a concern of migrant workers, which could be a solid reason for not utilizing preventive measures such as ITN. As a result, the current study’s low perceived severity of VL could be attributed to Eskilsson et al’s [53] previous observation: “If a disease is seen, as usual, it might not be severe” [53]. According to the migrant workers, VL was regarded as less severe because it was not commonly reported or mentioned by many of them. This would imply that, in terms of HBM factor susceptibility, perception of how common an illness is may influence the view of the disease’s seriousness, influencing the likelihood of taking preventive action, such as using bed nets.

### Perceived benefits and barriers

Even where vulnerability to VL was expressed, and despite the widespread belief that ITNs are an adequate measure of disease protection [31, 32, 54, 55], most respondents reported that they did not use bed nets because they are inconvenient and impossible to use all of the time due to their communal and outdoor sleeping arrangements on the farms. During harvesting season, for example, almost all migrant workers spend the night working in an open field inside the farm, rendering ITN use infeasible. This is consistent with a published literature review that identified common nighttime activity categories in low-income countries across different contexts involving recurring social and community events [32, 51, 52]. As a result, ITNs are not appropriate for this risk group, at least not during the harvesting season. Alternative solutions such as local neem oil, odorless neem, impregnated socks, long-lasting commercial skin repellent, and commercial wristbands should be considered. It should also be investigated to provide photographic images of people using bed nets in farmlands or other difficult settings. To that end, personal protection measures that have been shown to be effective should be promoted among those who must stay on the farmland at night to work or perform other duties [33–35, 51, 52].

Another major impediment to the use of ITN that emerged was the seasonal heat, particularly during harvesting activities. This finding is consistent with a review of the literature, which found that discomfort, primarily due to heat, was a major impediment to bed net use [26, 51, 52]. Although an improved *Gebaza* design that increases airflow may help reduce heat in the sleeping area, more research is needed on this topic.

The employees appeared to hold several incorrect beliefs about how VL is contracted. Aside from sandflies, VL was perceived to be caused by sexual intercourse, open defecation, messy environmental factors, filthy drinking water, bad food, and ravenousness, and prevention of these incorrect causes might decrease the perceived benefit of effective sandfly control measures (ITNs) [14, 22, 46, 51, 52, 56]. While these other causes will not result in VL, they may cause other diseases with similar signs and symptoms, such as Acute Watery Diarrhea (AWD). As a result, workers may take additional precautions, such as frequent handwashing, to protect themselves from VL. Interestingly, migrant workers were more confused about disease causes than key informants, indicating a serious deficiency in health education messages delivered by health professionals, resulting in a muddle of biomedical information.

### Intervention modality

For decades, VL control strategies have been largely ignored by researchers and funding agencies [57]. VL prevention and control should be considered for inclusion in integrated vector management in this regard. The findings of the current qualitative study will be of value for policymakers and program implementers interested in designing appropriate intervention programs against VL. The misperceptions identified with VL and sand flies should drive further research to develop alternative interventions and protective measures against VL [9].

The current study sends a clear message: no successful intervention program can be implemented unless misconceptions about the disease and sandflies are addressed. These misconceptions have a significant negative impact on actual behavior when it comes to using protective measures. As a result, it is critical to improving workers’ perceptions of the high risk and susceptibility to VL.

### Limitation

This study has a number of limitations that should be considered. First, there were some logistical issues that arose during the focus group discussions. The groups were formed based on the study participants’ availability of time. This frequently resulted in heterogeneous groups that did not freely converse during the discussion. Second, while the findings may be applicable to other comparative settings, generalization may be difficult due to the purposeful nature of the sampling technique. Nonetheless, the findings highlight issues that should be investigated further in order to develop better VL prevention strategies in the future. Third, our findings aim to provide a comprehensive understanding of migrant workers’ risk perception and use of vector control protective measures amongst this target group. We used a qualitative content analysis method that was guided by HBM. This could be criticized because it has the potential to bias the analysis and thus the study findings [34]. To ensure a non-biased approach to coding, we coded all transcripts inductively first and used the HBM to illuminate the later stages of the analysis. Finally, one of the study’s limitations was the demographics of the participants. Almost all of the participants were men due to the nature of the job. Female participants, if they were discovered, may have responded differently. The expertise of the researchers, who were able to encourage discussions and deal with issues through careful transcription and translation of the material collected in the FGDs, overcame the majority of the study’s limitations.

### Implications for health care

Increasing risk perception and knowledge of VL to influence the behavior of migrant workers is essential in promoting protective measures amongst this group. While there is widespread acceptance of the use of ITN, there is still a reluctance to implement protective measures. This is due to the low perceived seriousness, susceptibility, other barriers, and lack of knowledge [9] about VL and the protective measures demonstrated by the study’s FGD. The HBM used in this study [27] can help to promote risk perception and the use of protective measures among migrant workers.

### Implications for future research

While this study provides many valuable perceptions that can be considered when implementing VL prevention interventions, there may be other factors that influence the consistent use of VL preventive measures that are not influenced by individual perceptions. As a result, future research should consider factors other than individual perceptions to gain insight into how they influence intentions to use VL preventive measures. Furthermore, potential studies that investigate the variation in individual perceptions across seasons are required.

## Conclusion

The HBM was used in this study to examine factors that predict behaviors related to the use of measures that can reduce exposure to sandfly bites and thus VL transmission in Metema-Abuderafie lowland areas. Understanding migrant workers’ perceptions of perceived threat (susceptibility and severity), the likelihood of action (benefit and barrier), self-efficacy, and cues to action may improve prevention efforts. Overall, the study found that seasonal and migrant workers have a low perception of VL risk and the acceptability of measures to reduce sandfly bite exposure. It is recommended that well-designed programs be launched to increase migrant workers’ knowledge of VL and promote behavioral change that encourages them to adopt preventive behaviors. Other interventions are also required, such as assistance in obtaining ITNs and hanging them, as well as alternative protective measures for times when they are at risk from sandfly bites but would not use an ITN (like when working in the fields at night to avoid the heat of the day).

## Supplementary Information

**Additional file 1.** 

## Data Availability

The datasets used and analyzed during the current study are available from the corresponding author on reasonable request.
