# Supplementary material for: Understanding the risk perception of visceral leishmaniasis exposure and the acceptability of sandfly protection measures among migrant workers in the lowlands of Northwest Ethiopia: a health belief model perspective
Source: BMC Public Health. 2022 May 16;22:989. doi: 10.1186/s12889-022-13406-3 (PMC9112482; doi:10.1186/s12889-022-13406-3)
Supplement: Supplementary file 1 — Additional file 1. [file 12889_2022_13406_MOESM1_ESM.docx]

**Supplementary Data File 1.**

**Consolidated criteria for reporting qualitative studies (COREQ):**  **32-item checklist.**

Developed from: Tong A, Sainsbury P, Craig J. Consolidated criteria for reporting qualitative research (COREQ): a 32-item checklist for interviews and focus groups. International Journal for Quality in HealthCare.;19(6):349–57. Available from: http://www.ncbi.nlm.nih.gov /pubmed /17872937.

| **No. Item** | **Guide questions/description** | **Reported on Page #** |
| --- | --- | --- |
| **Domain 1: Research team and reflexivity** |  | |
| *Personal Characteristics* |  |  |
| 1. Interviewer/facilitator | Which author/s conducted the interview or focus group? | Resom Berhe, Adane nigusie, Zemekale Legese, and Mekuriawu Alemayehu |
| 2. Credentials | What were the researcher's credentials? E.g. PhD, MD | **Resom Berhe**  BSc, MPH  **Mark Spigt**  MSc, PhD, Associate Professor  **Mekuriaw Alemayehu**  **MPH, PhD, Associate Professor**  **Lucy Paintain**  MPH, PhD, Assistance professor  **Cherinet Adera**  MPH, MD  **Adane Nigusie**  MPH, PhD candidate  **Zemichael Gizaw**  MPH, PhD candidate  **Dia-Eldin A. Elnaiem**  MSc, PhD, Professor |
| 3. Occupation | What was their occupation at the time of the study? | Researcher at Gondar University, Maastricht University, LSHTM, Kala CORE, the Marry land University |
| 4. Gender | Was the researcher male or female? | Male reported in Methods |
| 5. Experience and training | What experience or training did the researcher have? | Methods -  The researcher  has a certificated in Qualitative Mixed Methods in international health from Emory University in 2017. |
| *Relationship with participants* |  | |
| 6. Relationship established | Was a relationship established prior to study commencement? | No |
| 7. Participant knowledge of the interviewer | What did the participants know about the researcher? e.g. personal goals, reasons for doing the research | Participant information sheet and Consent Form |
| 8. Interviewer characteristics | What characteristics were reported about the interviewer/facilitator? e.g. bias, assumptions, reasons and interests in the research topic | Methods |
| **Domain 2: study design** |  | |
| *Theoretical framework* |  |  |
| 9. Methodological orientation and Theory | What methodological orientation was stated to underpin the study? e.g.  grounded theory, discourse analysis, ethnography, phenomenology, content analysis | Methods |
| *Participant selection* |  | |
| 10. Sampling | How were participants selected? e.g. purposive, convenience, consecutive, snowball | Methods |
| 11. Method of approach | How were participants approached? e.g. face-to-face, telephone, mail, email | Methods |
| 12. Sample size | How many participants were in the study? | Methods |
| 13. Non-participation | How many people refused to participate or dropped out? Reasons? | Methods |
| *Setting* |  | |
| 14. Setting of data collection | Where was the data collected? e.g.  home, clinic, workplace | Methods |
| 15. Presence of nonparticipants | Was anyone else present besides the participants and researchers? | Methods |
| 16. Description of sample | What are the important characteristics of the sample? e.g. demographic data, date | Methods |
| *Data collection* |  | |
| 17. Interview guide | Were questions, prompts, guides provided by the authors? Was it pilot tested? | Methods |
| 18. Repeat interviews | Were repeat inter views carried out? If yes, how many? | No |
| 19. Audio/visual recording | Did the research use audio or visual recording to collect the data? | Methods |
| 20. Field notes | Were field notes made during and/or after the interview or focus group? | Methods |
| 21. Duration | What was the duration of the inter views or focus group? | Methods |
| 22. Data saturation | Was data saturation discussed? | Methods |
| 23. Transcripts returned | Were transcripts returned to participants for comment and/or correction? | Methods |
| **Domain 3: analysis and findings** |  | |
| *Data analysis* |  |  |
| 24. Number of data coders | How many data coders coded the data? | Methods |
| 25. Description of the coding tree | Did authors provide a description of the coding tree? | Methods |
| 26. Derivation of themes | Were themes identified in advance or derived from the data? | Methods |
| 27. Software | What software, if applicable, was used to manage the data? | Methods |
| 28. Participant checking | Did participants provide feedback on the findings? | Methods |
| *Reporting* |  | |
| 29. Quotations presented | Were participant quotations presented to illustrate the themes/findings? Was each quotation identified? e.g. participant number | Results |
| 30. Data and findings consistent | Was there consistency between the data presented and the findings? | Discussion |
| 31. Clarity of major themes | Were major themes clearly presented in the findings? | Results |
| 32. Clarity of minor themes | Is there a description of diverse cases or discussion of minor themes? | Results |
